# Global research trends and foci of artificial intelligence-based tumor pathology: a scientometric study

**DOI:** 10.1186/s12967-022-03615-0

**Published:** 2022-09-06

**Authors:** Zefeng Shen, Jintao Hu, Haiyang Wu, Zeshi Chen, Weixia Wu, Junyi Lin, Zixin Xu, Jianqiu Kong, Tianxin Lin

**Affiliations:** 1 Department of Urology, Sun Yat-Sen Memorial Hospital, Sun Yat-Sen University, Guangzhou, Guangdong China; 2 Graduate School of Tianjin Medical University, No. 22 Qixiangtai Road, Tianjin, 300070 China; 3 Zhujiang Hospital, Southern Medical University, 253 Gongye Road M, Guangzhou, 510282 China; 4 Guangdong Provincial Key Laboratory of Malignant Tumor Epigenetics and Gene Regulation, Guangzhou, China

**Keywords:** Bibliometric analysis, Artificial intelligence, Tumor, Pathology, VOSviewer, Citespace

## Abstract

**Background:**

With the development of digital pathology and the renewal of deep learning algorithm, artificial intelligence (AI) is widely applied in tumor pathology. Previous researches have demonstrated that AI-based tumor pathology may help to solve the challenges faced by traditional pathology. This technology has attracted the attention of scholars in many fields and a large amount of articles have been published. This study mainly summarizes the knowledge structure of AI-based tumor pathology through bibliometric analysis, and discusses the potential research trends and foci.

**Methods:**

Publications related to AI-based tumor pathology from 1999 to 2021 were selected from Web of Science Core Collection. VOSviewer and Citespace were mainly used to perform and visualize co-authorship, co-citation, and co-occurrence analysis of countries, institutions, authors, references and keywords in this field.

**Results:**

A total of 2753 papers were included. The papers on AI-based tumor pathology research had been continuously increased since 1999. The United States made the largest contribution in this field, in terms of publications (1138, 41.34%), H-index (85) and total citations (35,539 times). We identified the most productive institution and author were Harvard Medical School and Madabhushi Anant, while Jemal Ahmedin was the most co-cited author. *Scientific Reports* was the most prominent journal and after analysis, *Lecture Notes in Computer Science* was the journal with highest total link strength. According to the result of references and keywords analysis, “breast cancer histopathology” “convolutional neural network” and “histopathological image” were identified as the major future research foci.

**Conclusions:**

AI-based tumor pathology is in the stage of vigorous development and has a bright prospect. International transboundary cooperation among countries and institutions should be strengthened in the future. It is foreseeable that more research foci will be lied in the interpretability of deep learning-based model and the development of multi-modal fusion model.

**Supplementary Information:**

The online version contains supplementary material available at 10.1186/s12967-022-03615-0.

## Background

Traditional pathological examination is often realized by microscopy. By observing the histomorphological characteristics of cells or tissues that have been paraffin-fixed and mounted on glass slides, well trained pathologists can achieve disease diagnosis and classification [1, 2]. To date, the assessment of histopathological slides by pathologists is still the gold standard for tumor diagnosis [3, 4]. However, in spite of following the same diagnosis principles, diagnosis interpretations stand for the subjective analysis of pathologists, showing the non-standardized and low-repeatable decision-making process. This is the reason why significant interobserver variation often occurs even among highly-trained pathologists, which seriously affects the accuracy of tumor diagnosis [5]. Therefore, it is urgent to find an objective and reproducible method to realize tumor diagnosis and improve the diagnostic accuracy.

With the rise of digital pathology (DP), DP has changed the practice of traditional pathology, including its application in medical education and clinical practice [6–8]. As whole-slide scanner has become more widespread and popular, most glass slides can be digitized into whole slide images (WSI) for storing and analyzing through a computer-aided method [9]. DP plays a crucial role in modern clinical practice and is also a great solution to overcome the challenges that traditional pathology faced, such as heavy workload or low diagnostic accuracy [10]. Moreover, the application of WSI makes it easier and convenient for pathologists to enable a digital workflow, so as to achieve telepathology and clinical practice, which potentially changes the way of tumor diagnosis [11, 12].

Artificial intelligence (AI) was proposed by McCarthy et al. in the 1950s [13]. Since then, AI has been rapidly evolved and been extensively used in different fields ranging from science and technology, finance and medicine. The medical image analysis field has been an important field of AI-based research [14]. Through the predictive analytics of AI-based CT/MRI or other medical images, physicians can make better diagnosis and therapy decisions [15]. In term of DP, the introduction of WSI allows for AI-based predictive analytics in histopathology and WSI serves as a major platform for the application of AI in DP. With the progress of algorithm and network technology, especially the emergence of machine learning and deep learning, AI has been widely applied in the subfield of DP, particularly in oncology and precision medicine [16]. Compared with traditional pathology, the whole glass slice images can be obtained by AI-based WSI over a short period of time, then quantitative and qualitative analysis on the images can be conducted through deep learning to faster and more accurately identify new histopathological features, which is helpful for pathologists and physicians to understand and predict the progress and prognosis of the disease, and carry out in-time treatment intervention, so as to optimize individualized treatment and realize precision medical treatment. Moreover, the application of artificial intelligence algorithm makes the pathological diagnosis process more rapid, automatic and standardized [8].

In view of the aspects described above, research on AI-based DP has gained more and more attention of researchers, particularly for tumor pathology, which is the most major branch of DP research [17–20]. However, the explosive growth in the number of publications in this field has made it increasingly difficult for most researchers to keep up with the latest research findings. To date, there are only a few reviews or meta-analysis to summarize a certain aspect of AI-based tumor pathology research, while some important information is ignored, such as the contributions of authors, institutions, and future research forefront or foci. Bibliometric analysis, as a method that can quantitatively and qualitatively analyze and visualize all the documents published in a certain research field, has been widely used in medical fields [21–24].

Therefore, to gain much deeper insight into the AI-based tumor pathology research, this study aimed to identify the most productive countries, institutions or authors, and make an overall knowledge structure of scientific publications on AI-based tumor pathology research from 1999 to 2021 by bibliometric analysis, so as to provide the current research foci or hotspots and help scholars who have or are about to devote to this field.

## Methods

### Database and searching strategy

The Science Citation Index Expanded (SCI-Expanded 1999- present) of Clarivate Analytics’S Web of Science Core Collection (WoSCC) is one of the most comprehensive and influential databases in interdisciplinary fields, containing extensive academic journals and literature, which is wildly used as the data source for bibliometric study. In this study, all the publications we obtained were retrieved and downloaded from WoSCC database on February 24, 2022. The literature searching was performed by two independent researchers to ensure the reliability and authenticity of results. The searching strategy was formulated with reference to previous researches and the searching strategy was shown as follows: topic = (“artificial intelligence” OR “robotic*” OR “expert* system*” OR “intelligent learning” OR “feature* extraction” OR “feature* mining” OR “feature* learning” OR “machine learning” OR “feature* selection” OR “unsupervised clustering” OR “image* segmentation” OR “supervised learning” OR “semantic segmentation” OR “deep network*” OR “bayes* network” OR “deep learning” OR “neural network*” OR “neural learning” OR “neural nets model” OR “artificial neural network” OR “data mining” OR “graph mining” OR “data clustering” OR “big data” OR “knowledge graph”) [25] AND topic = (cancer* OR tumor* OR tumour* OR oncology OR neoplasm* OR carcinoma*) AND topic = (“Pathology” OR “histopathology” OR “Digital pathology” OR “Whole slide imag*” OR “Virtual microscopy” OR “Digital microscopy” OR “Digital slide*” OR “Virtual slide*” OR “Telepathology” OR “Telemicroscopy” OR “Computational pathology” OR “Computer-aided pathology” OR “Digital imag* analysis” OR “Pathology imag*” OR “pathomic*” OR “urine cytology” OR “Histopathological image*” OR “pathological image*”) AND publication year = (1999 to 2021) AND Language = (English). The document types were limited to original articles or reviews. The information including titles, authors, keywords, citations, journals, institutions and references of the publications were saved in plain text format.

### Data extraction and analysis

First, the documents were imported to Citespace V (Version 5.8.R3, Drexel University, United States) to remove duplicates. Then the targeted documents were collected and imported to GraphPad Prism 8.0 or Microsoft Excel 2019 by two independent authors for quantitative analysis of top-cited/productive countries, journals, authors, institutions, articles and keywords. In addition, H-index, a metric for evaluating the scientific impact of authors’ scholarly output and performance, was also collected from WoSCC [26].

### Data visualization

In this study, bibliometric analysis and visualization were performed by Citespace V (Version 5.8.R3), VOSviewer (Version 1.6.16) and a free online platform (http://bibliometric.com). VOSviewer is a wildly applied bibliometric analysis tool, which provided three kinds of visualization maps including the network visualization, the overlay visualization and the density visualization [27]. In this research, we mainly adopted VOSviewer to conduct author-keywords co-occurrence analysis, co-authorship analysis of countries/regions, authors, institutions, and co-citation analysis of journals or references. The options and settings of VOSviewer are summarized in Additional file 1: Table S1. Citespace V is another citation visualization analysis software developed by Chen et al. [28, 29]. Compared with VOSviewer, Citespace pays special attention to the connections between knowledge fields. By detecting and monitoring the development and changes of knowledge, it can visually comprehend the research frontiers and hotspots in these fields, and then predict the future development prospects and potential research directions of these fields [30]. In our study, Citespace was utilized to accomplish co-citation analysis of authors and references, dual-map overlay of journals and citation burst of keywords or references.

## Results

### Global trends of publication outputs and citations

**Fig. 1:**
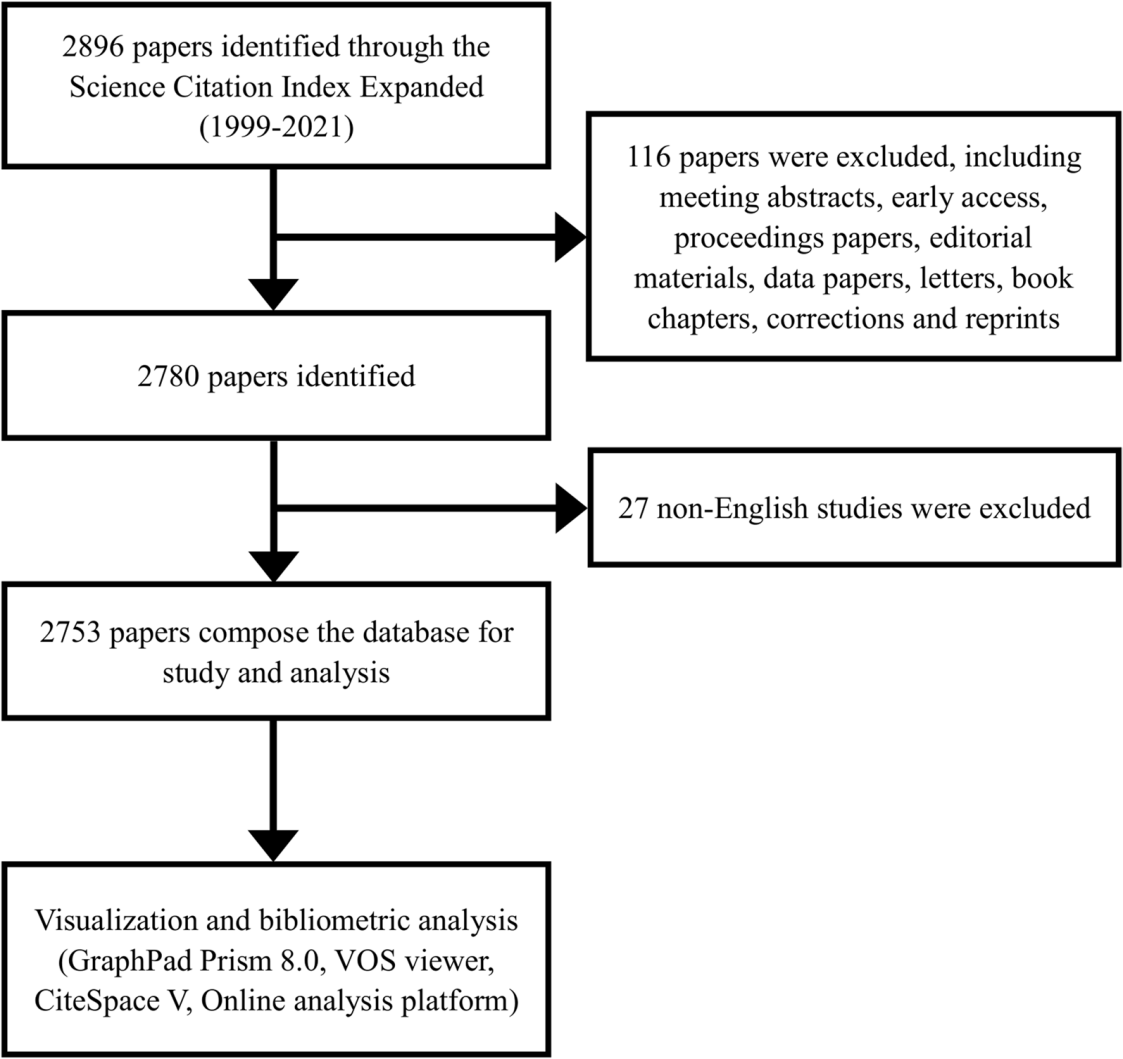
Flowchart of the publications selection in the study

**Fig. 2:**
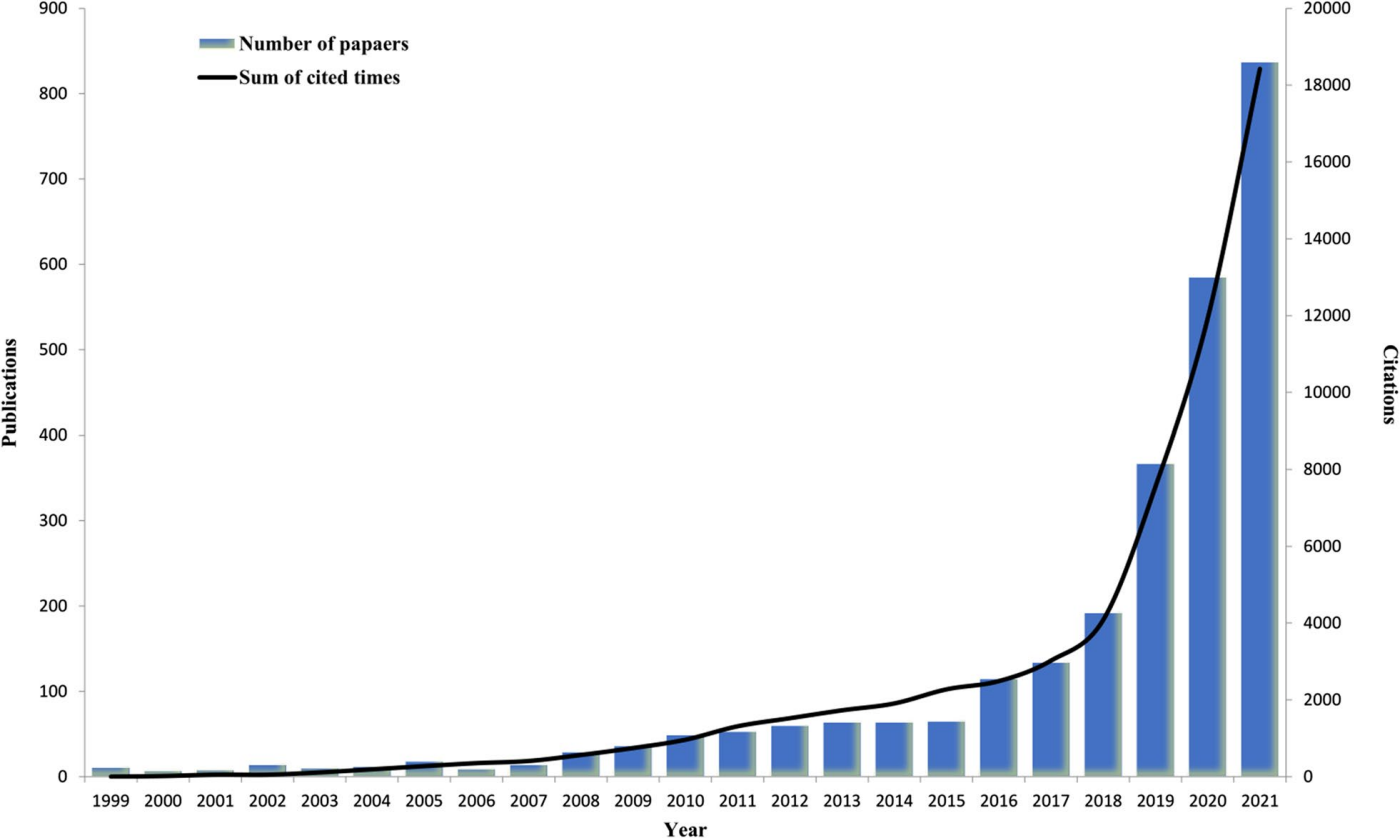
Global trend of publications and total citations on AI-based tumor pathology research over the past 23 years

The number of research papers published in different periods demonstrates the popularity and development tendency of research in a particular field. According to the search strategy and screening process, we collected 2753 literatures related to AI-based tumor pathology in recent 23 years from WoSCC (Fig. 1). As shown in Fig. 2, the number of research articles on AI-based tumor pathology research had been growing. Before 2007, there were only a few articles published. Then from 2008 to 2016, the amount of publications had increased, and after 2016, the number of publications began to grow exponentially, even reached 837 papers in 2021. As of the search date, the total citations of all literatures reached 62,182 times, and the average citations per paper and H-index were 22.59 and 101 respectively.

### Contributions of countries/regions

**Fig. 3:**
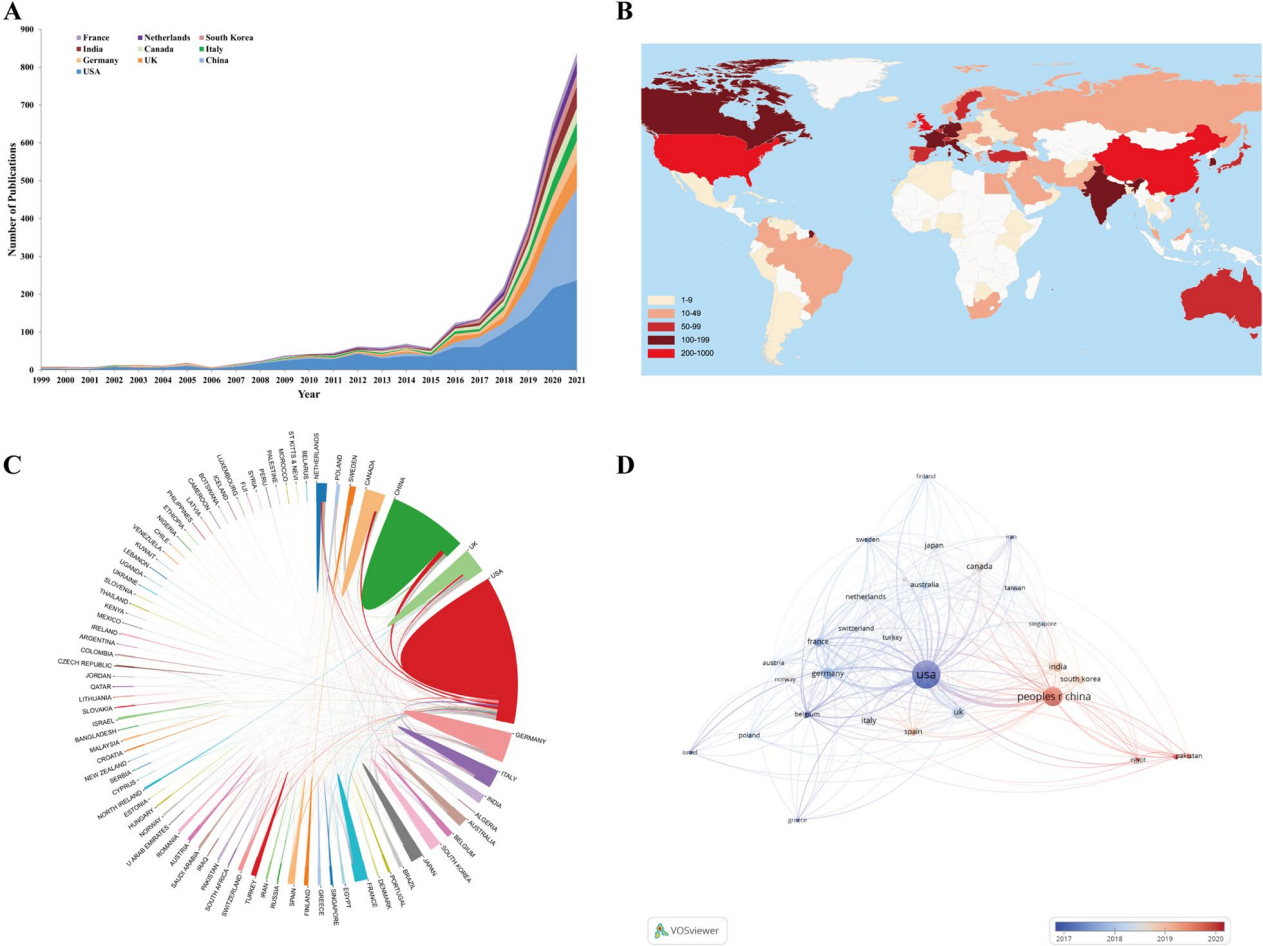
**A** The changing trend of the annual publication quantity in the top 10 countries/regions over the past 23 years. **B** Geographic distribution map based on the total publications of different countries/regions. **C** The cross-country/region collaborations visualization map. The thickness of the line between countries reflects the frequency of the cooperation. **D** The countries/regions citation overlay visualization map generated by using VOS viewer

A total of 86 countries/regions had contributed in the field of AI-based tumor pathology. Figure 3A showed the exponential publication changes of the top 10 countries from 1999 to 2021. Table 1 listed the top 10 productive countries in this field and it was obvious that the United States ranked first with 1138 (41.34%) publications, far more than twice that of China (541, 19.65%). Moreover, H-index and total citations of the United States were several times ahead of any other country with 85 and 35,539 respectively, indicating that the United States was the most cutting-edge country in this field in the world. The world map in Fig. 3B showed that publications in this field were mainly published by countries from North America, East Asia and Western Europe. Meanwhile, apart from the United States, countries with more than 200 publications also included China and the United Kingdom.

**Table 1:** Top 10 productive countries/regions in AI-based tumor pathology research

Rank	Country	Counts	Percentage	H-index	Total citations	Average citation per paper	TLS
1	USA	1138	41.34%	85	35,539	31.23	836
2	China	541	19.65%	36	5955	11.01	292
3	UK	242	8.79%	38	7234	29.89	423
4	Germany	187	6.79%	33	6648	35.55	365
5	Italy	158	5.74%	29	4109	26.01	292
6	Canada	154	5.59%	34	5836	37.90	247
7	India	153	5.56%	24	4021	26.28	114
8	South Korea	111	4.03%	22	1919	17.29	126
9	Netherlands	110	3.96%	31	7981	72.56	299
10	France	106	3.85%	30	4937	46.58	241

The international cooperation analysis was shown in Fig. 3C. It could be found that the cooperation among productive countries/regions was closely matched. As the most productive country, the United States cooperated closely with China, Germany and the United Kingdom. However, there was relatively little cooperation among other countries, especially the developing countries. As shown in Fig. 3D, 30 countries/regions were included and displayed. Among them, the top three countries/regions with the largest total link strength (TLS) were the United States (TLS = 836), the United Kingdom (TLS = 423), Germany (TLS = 365). In addition, the United States was the first country to start AI-based tumor pathology research, with an average publishing year of 2017.00, while the average publishing year of China was 2019.72.

### Contributions of top institutions and funding agencies

**Fig. 4:**
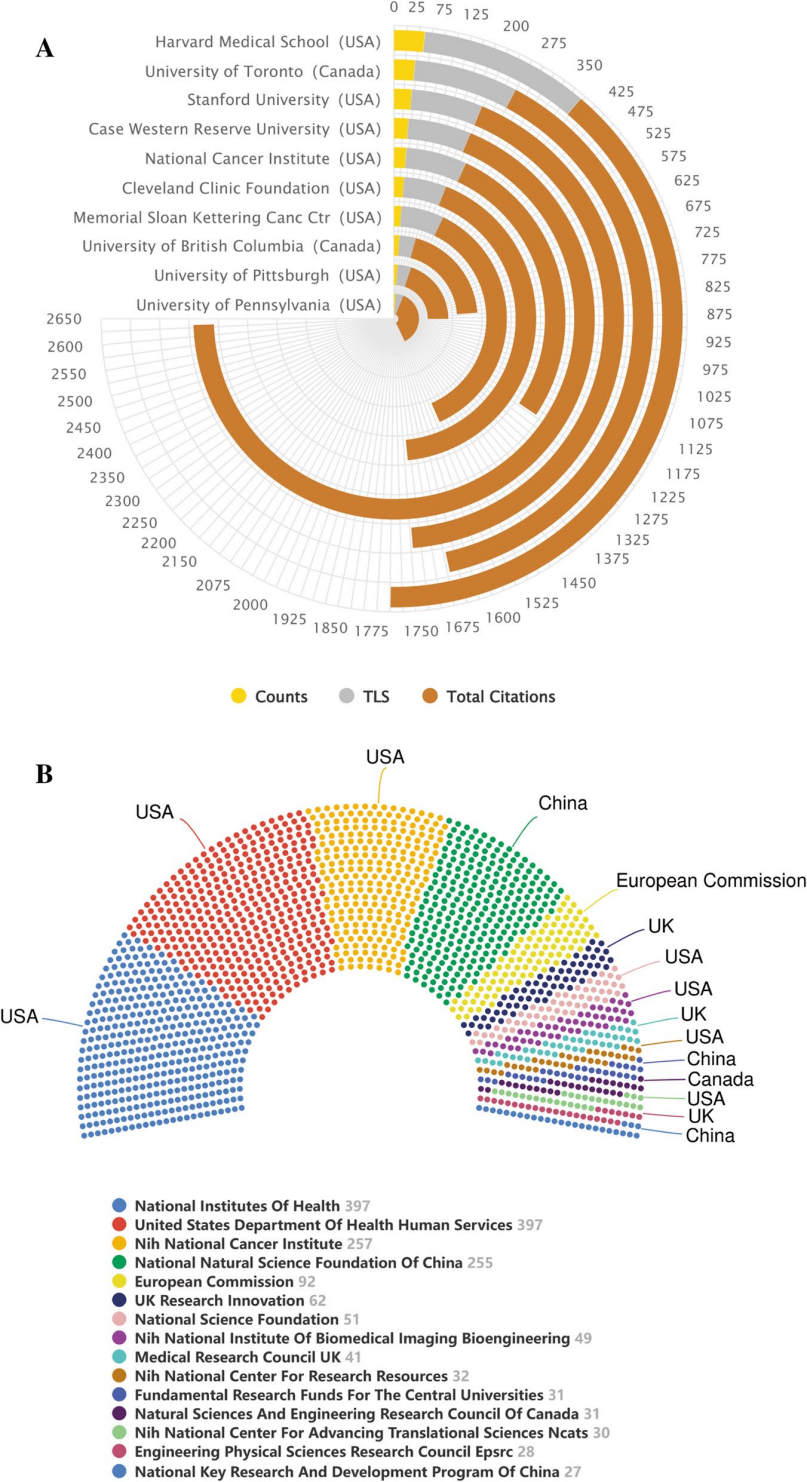
**A** The polar bar chart of counts, total link strength (TLS), total citations of the top productive 10 institutions. **B** The top most active funding agencies in AI-based tumor pathology research

More than 3600 institutions participated in publishing research papers on AI-based tumor pathology. The polar bar chart in Fig. 4A summarized the counts, TLS and total citations of the top productive 10 institutions in detail. It was evident that all the institutions in the top 10 were from North America, of which 8 were from the United States and 2 were from Canada. Specifically, Harvard Medical School ranked first with 61 papers, followed by the University of Toronto and Stanford University. In terms of TLS, Harvard Medical School, University of Toronto and Memorial Sloan Kettering Cancer Center ranked in the top three with 335, 229, 217 respectively. The top three institutions with the highest total citations were Case Western Reserve University (2413), Case Western Reserve University (1502) and Stanford University (1500). The result of Additional file 1: Fig. S1 showed that no organization had a betweenness centrality (BC) value higher than 0.1 and the density of the network map was low. In addition, Additional file 1: Fig. S2, an overlay visualization map generated by VOSviewer, showed that most institutions from North America or Europe, such as the University of Pennsylvania, Johns Hopkins University and Case Western Reserve University, entered the field earlier, while almost all Chinese institutions participated in this field after 2019 (the nodes were reddish).

Funding agencies played a key role in the conduct of research and the publication of articles. In the sight of that, Fig. 4B summarized the top 15 funding agencies by publications. From the results, there were a total of 7 funding agencies from the United States, of which the National Institutes of Health, United States Department of Health Human Services and NIH National Cancer Institute occupied the top three in this field. This result clearly demonstrated that the United States' leading position in this field was closely related to its strong economic foundation and support.

### Analysis of top journals and co-cited journals

**Fig. 5:**
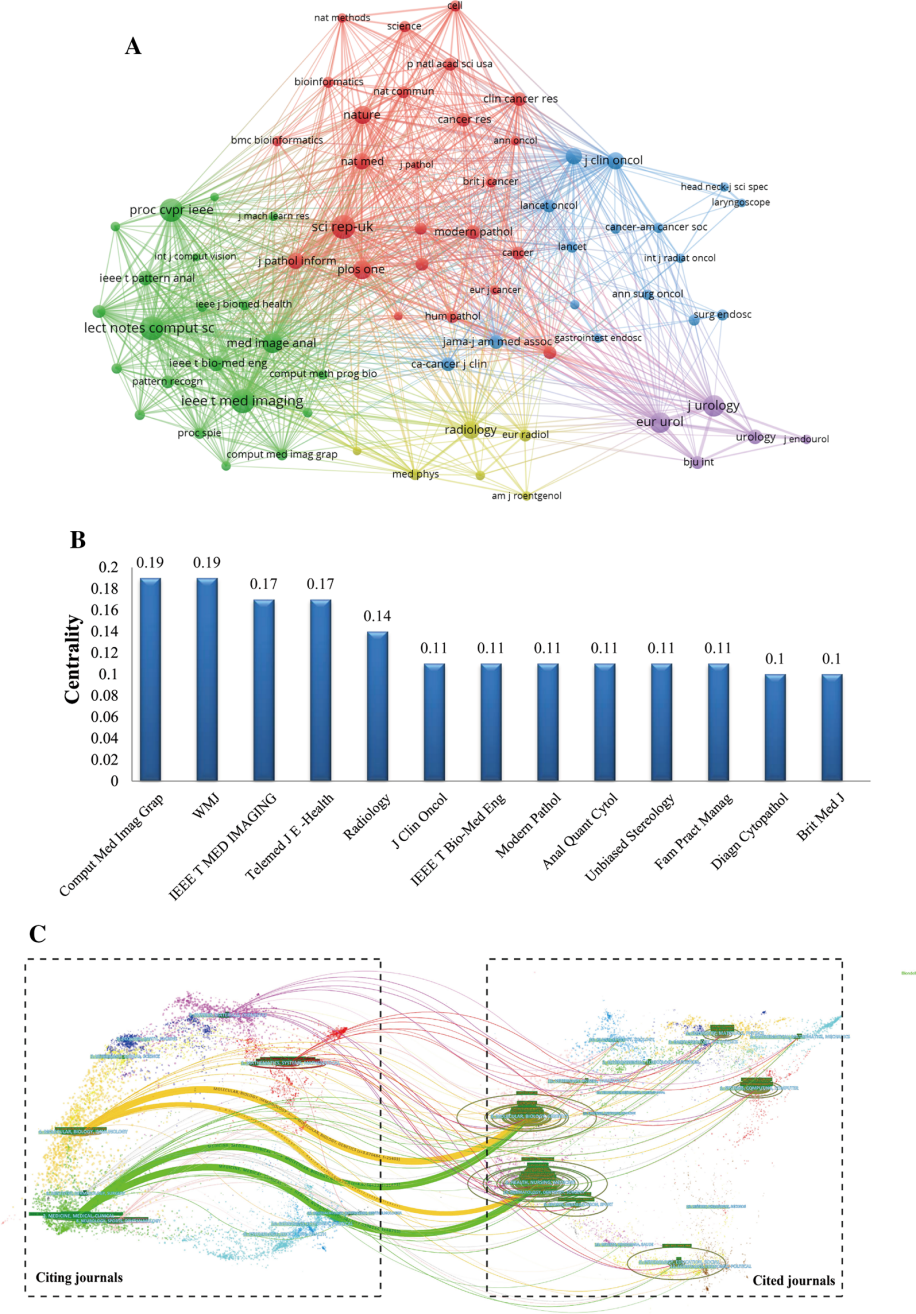
**A** Network visualization map of Journal co-cited analysis generated by VOSviewer. **B** Journal with a betweenness centrality value of no less than 0.1 (Journal co-citation analysis). **C** A dual-map overlap of journals on AI-based tumor pathology research carried out by Citespace

At present, the research papers related to AI-based tumor pathology had been published in 763 scholarly journals. From the results of Table 2, the journal *Scientific Reports* had the highest number of publications, with a total citations of 1537 times, followed by *IEEE Access* and *Frontiers in Oncology*. The total citations of *Medical Image Analysis* were 5491 times, which was higher than that of any other journals. Moreover, it was worth noting that although *European Urology* ranked 10th, its H-index (24) and total citations (2724 times) were much better than most journals listed. According to the 2020 Journal Citation Report (JCR), all the top 10 journals were located in Q1/Q2, and among them, *European Urology* (IF = 20.096) had the highest impact factor (IF). Journal co-citation is a significant index to reflect the influence of a journal. In this study, 73 journals had been co-cited at least 300 times and we used VOSviewer to generate a journal co-cited network map (Fig. 5A). As shown in Fig. 5A, the top three journals with the highest TLS were *Lecture Notes in Computer Science*, *IEEE Transactions on Medical Imaging*, and *Scientific Reports*. Figure 5B summarized the journals with BC value no less than 0.1, indicating the important role of these journals in this field. *Computerized Medical Imaging and Graphics* and *WMJ* had the largest BC value (0.19) and ranked first among all journals, followed by *IEEE Transactions on Medical Imaging* (0.17) and *Telemedicine Journal and e-Health* (0.17).

**Table 2:** Top 10 Journals related to the research of AI-based tumor pathology

Rank	Journal title	Countries	Counts	IF (2020)	JCR (2020)	H-index	Total citations
1	Scientific Reports	UK	87	4.38	Q1	20	1537
2	IEEE Access	USA	64	3.367	Q2	11	472
3	Frontiers in Oncology	Switzerland	55	6.244	Q2	7	175
4	CANCERS	Switzerland	52	6.639	Q1	9	293
5	Medical Image Analysis	Netherlands	50	8.545	Q1	20	5491
6	IEEE Transactions on Medical Imaging	USA	47	10.048	Q1	21	2382
7	IEEE Journal of Biomedical and Health Informatics	USA	34	5.772	Q1	10	300
8	BJU International	UK	32	5.588	Q1	21	1061
9	Computers in Biology and Medicine	USA	30	4.589	Q1/Q2	11	404
10	European Urology	Netherlands	28	20.096	Q1	24	2724

Figure 5C was a dual-map, which was used to represent the discipline distribution of journals involved in AI-based tumor pathology research, and through this method, we could clearly understand the knowledge flows among different disciplines and the frontier or hotspot of each discipline. it could be found that the literature published in Molecular/Biology/Immunology or Medicine/Medical/Clinical journals often cited the literature from Molecular/Biology/Genetics or Health/Nursing/Medicine journals.

### Analysis of the active authors and co-cited authors

**Fig. 6:**
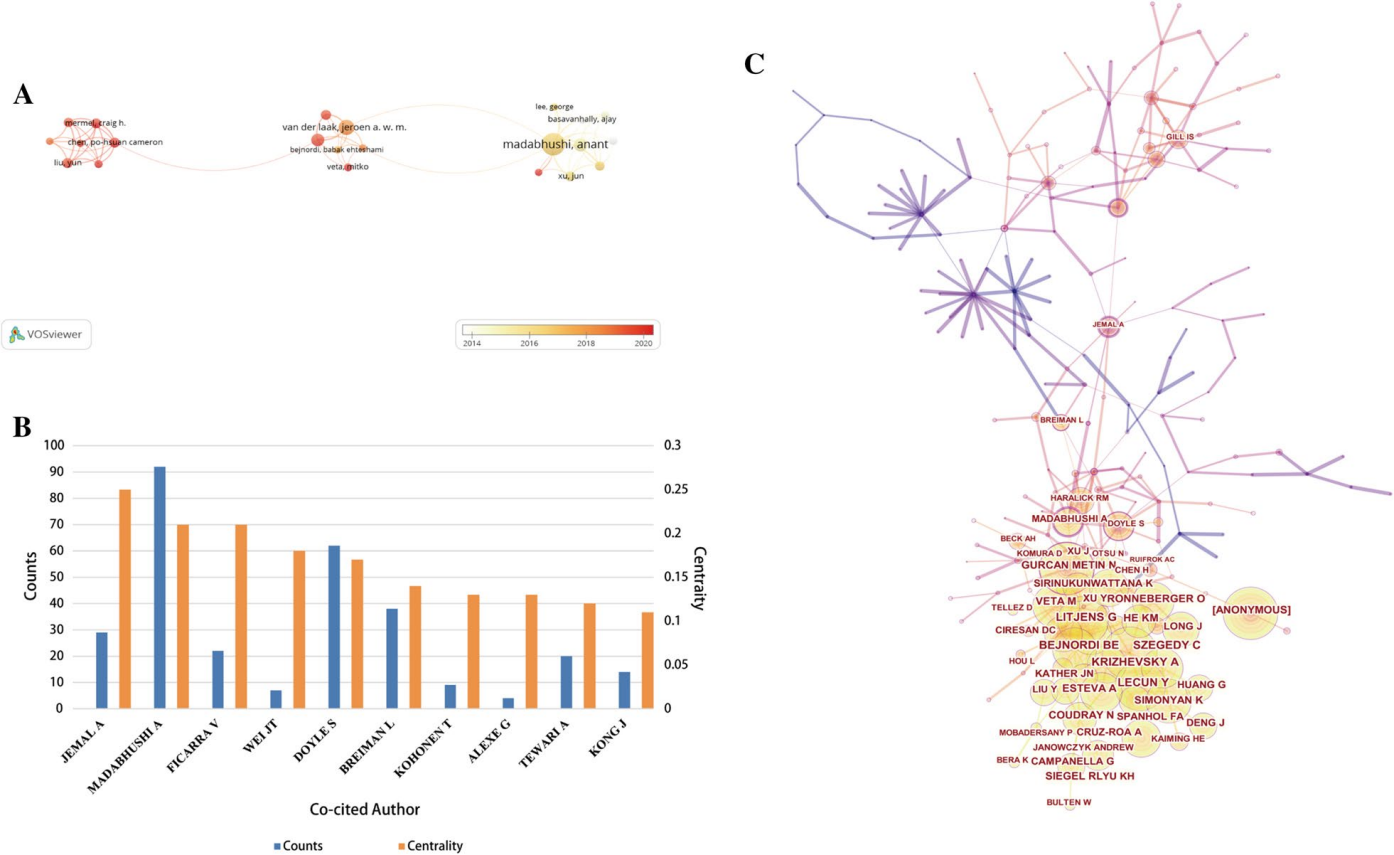
**A** The visualization map of author co-authorship analysis generated by VOSviewer. **B** Authors with a betweenness centrality value of more than 0.1 (author co-citation analysis). **C** The visualization map of author co-citation analysis produced by Citespcae

A total of 15,182 authors participated in the publication of papers in the field of AI-based tumor pathology. Table 3 summarized the top 10 most productive authors and the top 10 co-cited authors. The top 10 most productive authors were mostly from the United States and European countries. Madabhushi Anant, Rajpoot Nasir M and Yang Lin ranked in the top three with 40, 25 and 20 papers respectively. While Van Der Laak Jeroen A. W. M. and Litjens Geert from the Netherlands published fewer papers, their total citations were as high as 5230 and 5117, respectively. Figure 6A was a visualization map of author co-authorship analysis generated by VOSviewer. As shown in Fig. 6A, Van Der Laak Jeroen A. W. M. and Litjens Geert were key authors connecting multiple research clusters. However, overall, there was little collaboration and communication among various research clusters. Through co-citation analysis of authors, we found that the total citations of Bejnordi BE, Litjens Geert and Szegedy C ranked in the top three, and the total citations of the top 10 co-cited authors exceeded 240 times (Table 3). In this study, the BC values of 10 authors exceeded 0.1 (Fig. 6B). Among them, Jemal Ahmedin, Madabhushi Anant and Ficarra Vincenzo were the top three, up to 0.25, 0.21 and 0.21 respectively, showing their centrality in this field. Figure 6C was the map of author co-citation analysis produced by Citespcae. The results also demonstrated that Madabhushi Anant et al. were at the center of the research in this field.

**Table 3:** The 10 most productive authors and top 10 co-cited authors in AI-based tumor pathology research

Rank	Author	Country	Counts	Total Citations	Co-Cited Author	Country	Total Citations	TLS
1	Madabhushi, Anant	USA	40	2765	Bejnordi, BE	Netherlands	368	21,103
2	Rajpoot, Nasir M	UK	25	1011	Litjens, Geert	Netherlands	361	22,098
3	Yang, Lin	China	20	617	Szegedy, C	USA	330	18,504
4	Van Der Laak, Jeroen A. W. M	Netherlands	19	5230	Lecun, Yann	USA	325	19,669
5	Kaouk, Jihad H	USA	18	822	Krizhevsky, Alex	USA	311	18,006
6	Feldman, Michael	USA	15	1078	Veta, Mitko	Netherlands	309	19,552
7	Pantanowitz, Liron	USA	15	168	He, KM	China	306	17,054
8	Litjens, Geert	Netherlands	14	5117	Kather, Jakob Nikolas	Germany	288	18,142
9	Kather, Jakob Nikolas	Germany	13	243	Spanhol,Fabio Alexandre	Brazil	287	14,833
10	Pinto, Peter A	USA	12	512	Simonyan, Kristina	USA	247	14,512

### Analysis of references and co-cited references

**Fig. 7:**
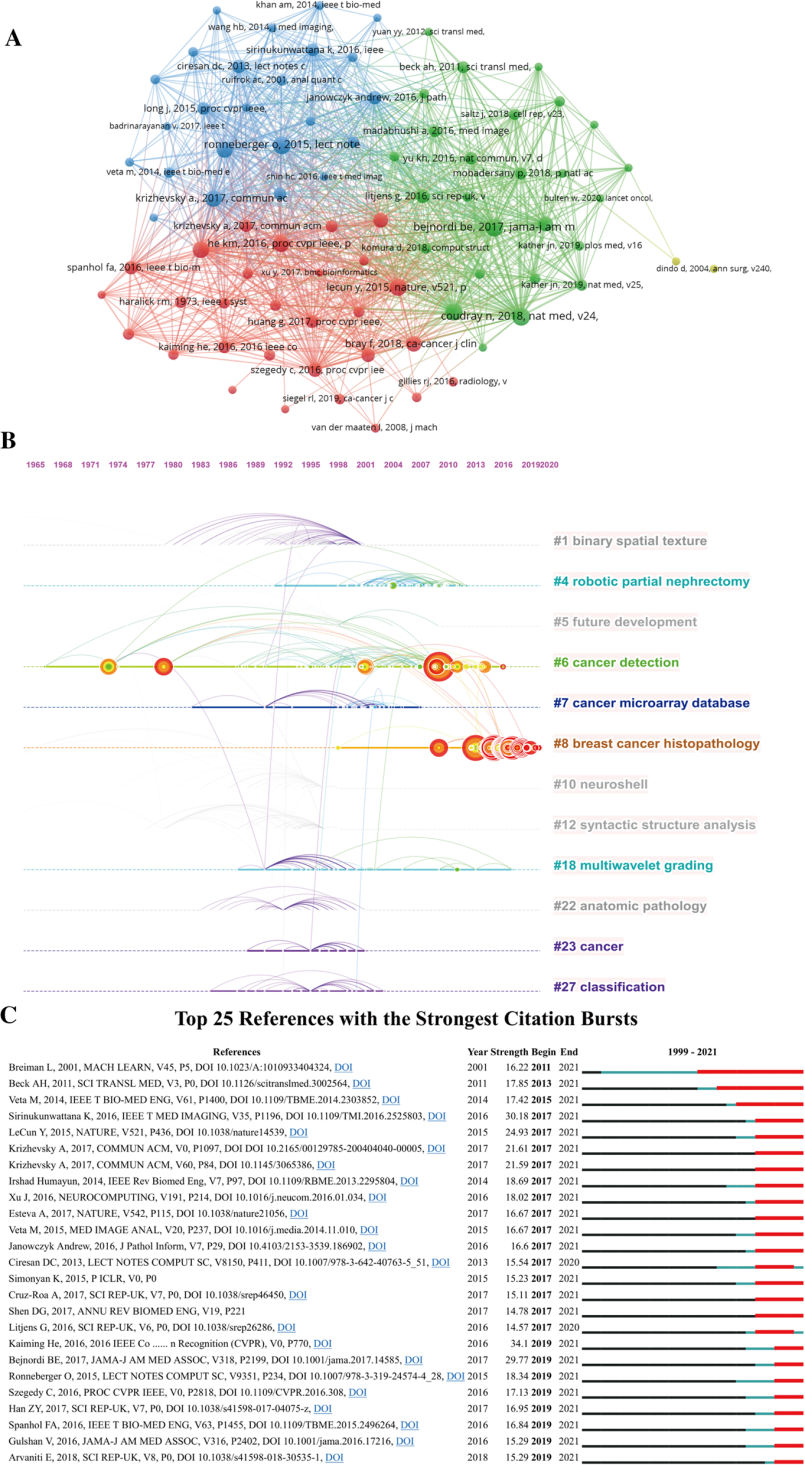
Network visualization map of Cluster view **A** and timeline view **B** of co-citation references. The time evolution is indicated with different colored lines and the nodes on the lines indicate the references cited. **C** Visualization map of top 25 references with the strongest citation bursts in AI-based tumor pathology research

A total of 2753 papers were included in this study, of which 102 publications were cited more than 100 times. Table 4 listed the top 10 cited papers and the most cited article was the review published by Litjens Geert in 2017, with a total of 3777 times, followed by Rhodes [31] and Tothill Richard W. (2008), with 2425 and 929 times respectively. As shown in Fig. 7A, [32–34] were the three most co-cited references, with a total of 221, 215, 214 citations respectively. The timeline view of co-citation reference is a visual diagram that can reflect the temporal characteristics of the research hotspots in this field. According to the results of Fig. 7B, the Modularity Q was 0.9545, and the mean Silhouette S was also as high as 0.9839, showing the excellent clustering effect and network homogeneity. Among the 12 clusters, #6 cancer detection was the earliest research hotspot in this field, and had been studied until recent years. To date, the most popular research hotspot is #8 breast cancer histopathology, and more researchers may pay more attention to these research foci. Top 25 references with the strongest Citation bursts were summarized in Fig. 7C. The reference citation burst in this study began in 2011 due to the paper published by Breiman Leo in 2001 [35]. The latest reference citation burst was detected in 2019 and last until now. Among them, the paper on new deep residual nets published by Kaiming He et al. [36] in 2016 had the strongest strength value. Currently, most important articles are still cited frequently and it can be speculated that AI-based tumor pathology research will still be a research hotspot in the next few years.

**Table 4:** Top 10 original articles concerning the research of AI-based tumor pathology

Title	Journals	First author	Year	citations
A survey on deep learning in medical image analysis	Medical Image Analysis	Litjens Geert	2017	3777
ONCOMINE: A cancer microarray database and integrated data-mining platform	Neoplasia	Rhodes DR	2004	2425
Novel molecular subtypes of serous and endometrioid ovarian cancer linked to clinical outcome	Clinical Cancer Research	Tothill Richard W	2008	929
Diagnostic Assessment of Deep Learning Algorithms for Detection of Lymph Node Metastases in Women With Breast Cancer	Journal of The American Medical Association	Bejnordi Babak Ehteshami	2017	899
Using Fourier transform IR spectroscopy to analyze biological materials	Nature Protocols	Baker Matthew J	2014	881
DNA methylation-based classification of central nervous system tumours	Nature	Capper David	2018	865
Computer-aided diagnosis in medical imaging: Historical review, current status and future potential	Computerized Medical Imaging and Graphics	Doi Kunio	2007	832
Gene expression-based classification of malignant gliomas correlates better with survival than histological classification	Cancer Research	Nutt CL	2003	697
Classification and mutation prediction from non-small cell lung cancer histopathology images using deep learning	Nature Medicine	Coudray Nicolas	2018	668
Locality Sensitive Deep Learning for Detection and Classification of Nuclei in Routine Colon Cancer Histology Images	IEEE Transactions on Medical Imaging	Sirinukunwattana Korsuk	2016	509

### Keywords co-occurrence analysis

**Fig. 8:**
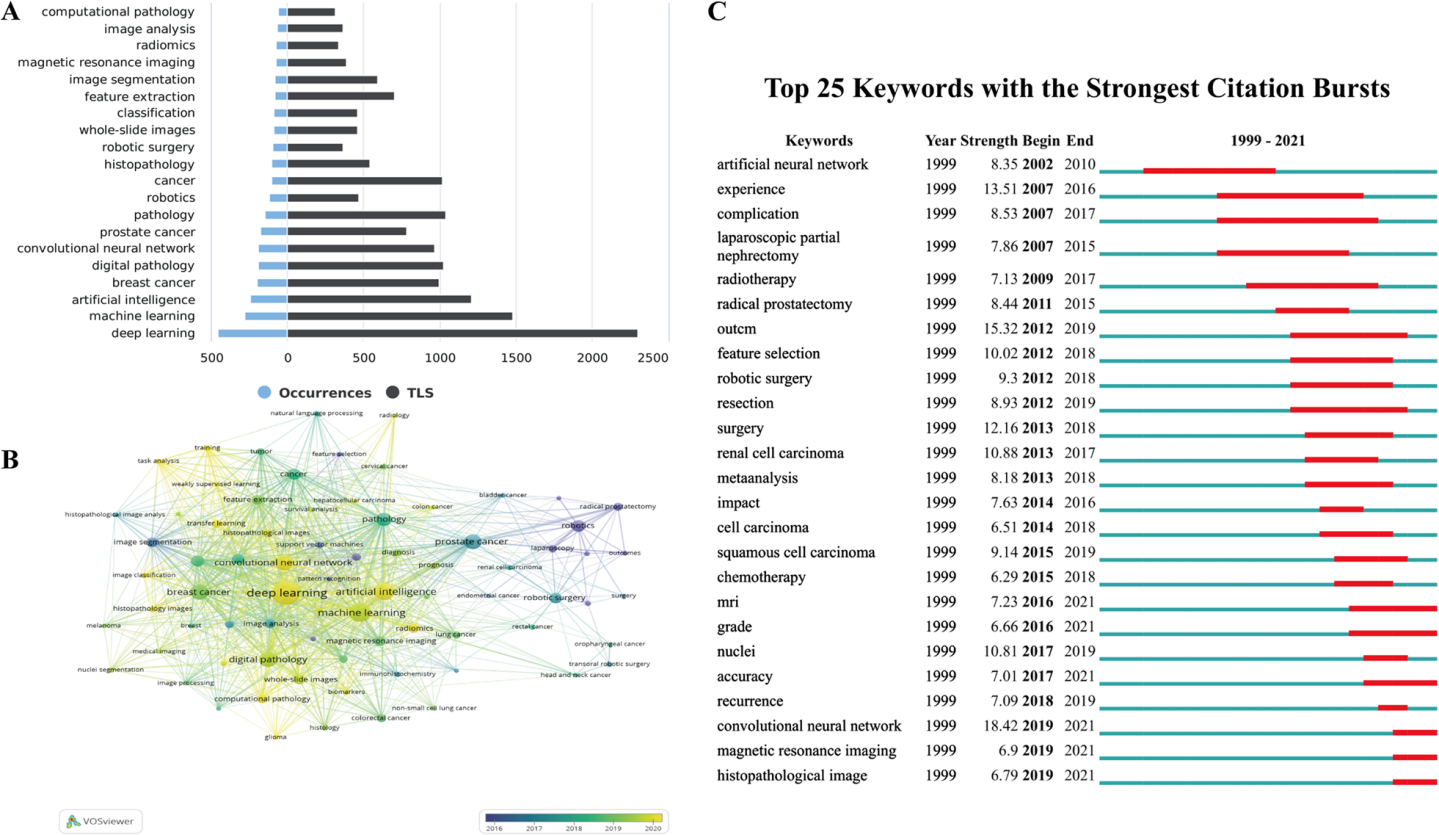
**A** The top 20 author keywords with the highest frequency. **B** The overlay visualization map of author keywords co-occurrence analysis. **C** Visualization map of top 25 keywords with the strongest citation bursts in AI-based tumor pathology research

A total of 5279 author keywords were included in this study. Figure 8A summarized the top 20 keywords with the highest frequency. The top three most common keywords were “deep learning”, “machine learning” and “artificial intelligence”, which was consistent with our study topic. In addition, “breast cancer” and “prostate cancer” were currently the most studied tumors in this field. In addition, the top 20 commonly investigated cancers/tumors were also listed in Additional file 1: Table S2. As shown in Fig. 8B, All keywords were marked with different colors depending on the temporal sequence of keyword appearance. It was noticeable that “deep learning” located in the central position of the visualization map. Moreover, except for “robotics”, “laparoscopy” and other surgery-related keywords, most of the keywords, such as “nuclei segmentation”, “computational pathology” or “transfer learning” appeared after 2019, indicating that the research in this field was rising in recent years. In other words, AI-based tumor pathology study is of high research value and will become a hot topic for a long time in the future.

The top 25 keywords with the strongest citation burst were listed in Fig. 8C. “Artificial neural network”, the earliest keyword burst, was detected in 2002. Later, researches related to tumor treatment such as “radiotherapy” or “robotic surgery” became hot topics. The latest keyword burst occurred in 2019 and had last until now. The major keywords were “convolutional neural network“, “magnetic resonance imaging” and “histopathological image”, suggesting that these research topics had received extensive attention in recent years and might become new research foci in the next few years.

## Discussion

In the era of explosive growth of information, it is very difficult to maintain sensitivity to research hotspots, master the latest research results and maintain a leading position in the research field. Therefore, bibliography retrieval and knowledge management are the routine tasks of every scientific researcher. Different from systematic review or meta-analysis, bibliometric analysis has the advantages of summarizing the development of specific research fields as well as analyzing research hotspots. This is the first study to summarize the application and development of AI-based tumor pathology through bibliometric methods, showing the development trend of AI-based tumor pathology in the past 23 years, and predicting future research hotspots in this field.

To a certain extent, the number of scientific articles reflect the development of research in a particular field. The results of this study showed that during 1999–2021, publications on AI-based tumor pathology had been increasing, especially in the past 6 years, the number of published papers accounted for 81% of all publications, which benefits from the sharp development of deep learning. In addition, the number of papers published has increased rapidly after 2016, mainly due to the proposal and application of a variety of new deep learning frameworks, such as deep residual networks, spatially constrained convolutional neural network (SC-CNN), etc. AI-based tumor pathology has become an important research field in clinical practice, and has a bright prospect.

According to the results of countries/regions distribution, among the 86 countries/regions involved in this study, the United States (1138, 41.34%) was the country with the largest number of published articles, followed by China (541, 19.65%), which together accounted for 60.99% of all papers, demonstrating their leadership in the study of AI-based tumor pathology. However, the total citations in China was unsatisfactory, especially the average citation per paper, which was the lowest among the top 10 countries/regions in terms of productivity (Table 1). China was the country with the fastest growth in the number of publications in this study, but it still lacked highly-cited or high-quality research, which leaded to its insufficient international influence. It can be seen from Fig. 3D that China, India and many other countries participated in the field of AI-based tumor pathology later than the United States, Canada and Germany, showing that they were newly active in this field and may have a more important position in the future.

As for countries/regions cooperation, the United States was the center of research and had close cooperation with China, Germany and the United Kingdom. However, most cooperation and research communication were limited to North America, Europe and a few Asian countries. Therefore, international transboundary cooperation was essential in the future, especially with developing countries/regions. It cannot be denied that economic support also plays an important role in supporting scientific research output. Increased investment of encouragement and funding support in scientific research may need in many countries, so that they may become important participants in this field in the future.

The top 10 productive institutions were all from North America, of which 8 belong to the United States and 2 were from Canada. Harvard Medical School was the most productive and influential institution, and it also maintained close cooperative relationships with multiple countries/regions, including institutions from China. However, although some institutions in China, such as Shanghai Jiao Tong University and Southern Medical University, had also published many papers and achieved a certain academic influence, there were not much close cooperation and exchanges with academic institutions in other countries. In addition, the BC value of all institutions was lower than 0.1, which suggested that research institutions in this field were scattered. Therefore, academic institutions in various countries needed to strengthen cooperation with each other, to further improve the academic status of the country.

Identification of important journals and journal co-citation analysis can provide researchers with a wealth of reliable reference information and is helpful for them to determine the most suitable target journals when searching for literature or submitting their research [37]. In addition to total citations, impact factor (IF) and JCR [38, 39] category are two important indicators for evaluating the academic status of journals. Most of the journals listed in Table 2 were comprehensive journals, mainly including oncology, medical imaging and AI. It could be found that all the top 10 journals located in Q1/Q2, and the IF ranged from 3.367 to 20.096, indicating that AI-based tumor pathology related articles could also be published in high-impact journals. *Scientific Reports* was the journal with the largest number published articles, showing that most articles related to this field would be considered for publication in this journal. Furthermore, it is worth noting that *BJU International* and *European Urology* both were important journal in urology, indicating that urogenital neoplasm was one of the hotspots in AI-based tumor pathology research.

Journal co-citation analysis provides insight into the connections between different research findings [40]. *Scientific Reports, Lecture Notes in Computer Science, IEEE Transactions on Medical Imaging* and *Medical Image Analysis* were the journals with TLS over 100,000, which indicated that the research papers related to AI-based tumor pathology in such journals were more likely to be cited. The results in Fig. 5B showed that *Computerized Medical Imaging and Graphics* and *WMJ* had the largest BC value (0.19). It is suggested that researchers in this field could pay more attention to the research findings published in these journals to obtain the latest research progress.

In author co-authorship analysis, five of the top 10 most active authors were from the United States and they published a total of 100 papers. Madabhushi, Anant from the United States contributed the most papers, followed by Rajpoot, Nasir M. from UK and Yang Lin from China with 25 and 20 papers respectively. A point worth noting was that although Van Der Laak Jeroen A. W. M. and Litjens Geert published few papers, their total citations exceeded 5000 times, indicating their important position in this field. Meanwhile, from Fig. 6A we found that Van Der Laak, Jeroen A. W. M. and Litjens Geert were also the critical authors connecting multiple research clusters, which may explain the reason for their high citations. However, the BC value for each author was lower than 0.1 in the author co-authorship analysis, which reflected the little cooperation between different research teams. Consequently, international transboundary cooperation should be strengthened.

As for author co-citation analysis, the BC values of Jemal Ahmedin, Madabhushi Anant and Ficarra Vincenzo reached 0.25, 0.21 and 0.21, respectively. Jemal Ahmedin is a well-known expert in the field of oncology and has published several Cancer statistics in the *CA-A Cancer Journal for Clinicians *[41, 42, 43]. Madabhushi Anant, who works at the Department of Biomedical Engineering in Case Western Reserve University, and his colleagues published a key paper using an instance of a deep learning strategy, Stacked Sparse Autoencoder (SSAE), paved the way for efficient nuclei detection on high-resolution histopathological images of breast cancer [44]. Ficarra Vincenzo is an expert in urology, focusing on the research of surgical treatment of urogenital cancer and many of his articles have been cited more than 200 times [45–48]. Therefore, we believe that in terms of the AI-based tumor pathology research, more important articles may be published by the above team members, strengthening cooperation with these top teams is a good choice for research.

Citation analysis and co-citation analysis of reference are important means in a bibliometric study, which use to identify important literature as well as evaluate the research evolution and predict the frontiers of research development. High-cited articles are usually high-quality research with strong innovation and significant impact in a certain field. Table 4 listed the top 10 most cited studies, all of which had more than 500 citations and have significant influence in this field. Specifically, the review of Litjens Geert, “A survey on deep learning in medical image analysis” published on *Medical Image Analysis* had been cited 3777 times, which was the most cited article in this field [49]. The article summarized the main deep learning concepts related to medical image analysis and multiple contributions to this field. Also, it discussed the state-of-the-art technology and future research foci of deep learning. Another article with more than 2400 citations was published in 2004 by Rhodes DR. His team demonstrated “ONCOMINE”, a cancer microarray database and web-based data-mining platform that facilitated the discovery of genome-wide expression analysis [31].

Burst detection is an algorithm developed for capturing the sharp increases of references or keywords popularity within a certain period, which can serve as an efficient method to identify hotspots or topics. Our findings suggested that the first reference citation burst in the field started in 2011 and continued until 2021. It was due to the research on Random Forests published by Breiman L in 2001 [35], which introduced a machine learning algorithm with more robustness to noise, and laid the foundation for a series of subsequent studies. Figure 7C showed that most of the reference citation burst were still in progress, and the latest one began in 2019, caused by multiple researches. Among them, the strongest strength value was the literature on new deep residual nets published by Kaiming He et al. in 2016 [36]. His research team introduced a new deep learning model to deal with deeper neural training and achieved good results, having a certain impact on visual recognition in the future.

Co-occurrence analysis of keywords is a common method used in bibliometrics to identify popular research topics, which can reflect the changing process of research topics in the whole field and better grasp the research hotspots [50]. As shown in Fig. 8A, “deep learning”, “machine learning” and “artificial intelligence” were the most frequently occurring keywords, which were consistent with the topic of this study. “Breast cancer” and “prostate” cancer” were the most keywords among all tumor keywords. To date, breast cancer is the cancer with the highest incidence among women, while prostate cancer is the second most common cancer in men, and both are currently the most common causes of cancer related death [51, 52]. How to achieve quick and accurate tumor staging or grading through pathology for precise treatment is the current research focus in this field. In addition, the combination of multiomics analysis [53–55] such as radiomics [56, 57] is the focus of future breakthrough in digital tumor pathology. Of course, this process requires more powerful algorithm updates and funding support.

Keywords burst detection in Fig. 8C showed that the first detected keyword was “artificial neural network” in 2002. from 2007 to 2019, keywords related to tumor treatment such as “radiotherapy”, “robotic surgery” or “chemotherapy” had become popular researches topics. The latest burst began in 2019, including the following keywords: “convolutional neural network”, “magnetic resonance image” and “histopathological image”. With the popularization of artificial intelligence and the renewal of deep learning algorithm, convolutional neural network has become the most important algorithm for processing medical images, especially in radiology and histopathology [58–60]. However, deep learning-based AI has been queried by both clinician and pathologists for the lack of good interpretability, hindering the clinical application of AI model [61–63]. Therefore, the development of interpretable deep learning algorithm is the focus of breakthrough for better application of deep learning-based AI in clinical practice. In addition, gone were the days of diagnosing or classifying diseases through a single pathological tissue section or radiological imaging. Many studies have shown that multimodal fusion methods, integrating proteomics, radiomics, genomics, etc. are much more accurate in tumor diagnosis, staging or prognosis prediction [64, 65]. The multi-modal fusion model may be also an important topic for the future development of tumor pathology.

## Limitations

There are some limitations worth noting in this study. First of all, we only selected WoSCC as our database, which indicates that we may miss some related papers in some other databases [66]. However, in the view of limitations of bibliometric software, it is difficult to merge various database for analysis and we also present the main reasons for choosing WoSCC as our database in Methods section. Secondly, it may overlook some significant non-English papers, resulting in research bias and decreased credibility. Finally, due to the continuous updating of database, recently published high-quality articles may be underestimated for their unsatisfactory citations [67, 68, 69].

## Conclusions

In summary, this is the first comprehensive analysis of publications related to AI-based tumor pathology from 1999 to 2021 through bibliometrics. Our results show that AI has been widely applied in tumor pathology and is still in sharp development, indicating that the research in AI-based tumor pathology will increase significantly in the future. To date, the United States still dominates the field of AI-based tumor pathology while China also evolves rapidly. Whether institutions or countries, international transboundary cooperation should be strengthened, especially for the Asian countries. In addition, breast cancer and prostate cancer are the most studied tumors at present. The key foci of AI-based tumor pathology research in the future lie in the interpretability of deep learning-based model and the development of multi-modal fusion model.

## Supplementary Information

**Additional file 1**: **Figure S1** The institutional cooperation map created with Citespace. **Figure S2** The overlay visualization map of institution co-authorship analysis generated by VOSviewer. **Table S1** The options and settings of VOSviewer for AI-based tumor pathology study. **Table S2** The top 20 commonly investigated cancers/tumors in the field of AI-based tumor pathology based on the frequency of author keywords co-occurrence.

## Data Availability

The datasets generated and/or analysed during the current study are available in the [The Science Citation Index Expanded (SCI-Expanded 1999- present) of Clarivate Analytics’S Web of Science Core Collection (WoSCC)] repository, https://www.webofscience.com/wos/alldb/basic-search.

## Abbreviations

AI: Artificial intelligence
DP: Digital pathology
WSI: Whole slide images
WoSCC: Web of Science Core Collection
IF: Impact factor
TLS: Total link strength
BC: Betweenness centrality
JCR: Journal Citation Report
