# Supplementary material for: Global research trends and foci of artificial intelligence-based tumor pathology: a scientometric study
Source: J Transl Med. 2022 Sep 6;20:409. doi: 10.1186/s12967-022-03615-0 (PMC9450455; doi:10.1186/s12967-022-03615-0)
Supplement: Supplementary file 1 — Additional file 1: Figure S1 The institutional cooperation map created with Citespace. Figure S2 The overlay visualization map of institution co-authorship analysis generated by VOSviewer. Table S1 The options and settings of VOSviewer for AI-based tumor pathology study. Table S2 The top 20 commonly investigated cancers/tumors in the field of AI-based tumor pathology based on the frequency of author keywords co-occurrence. [file 12967_2022_3615_MOESM1_ESM.docx]

**Additional File**

**
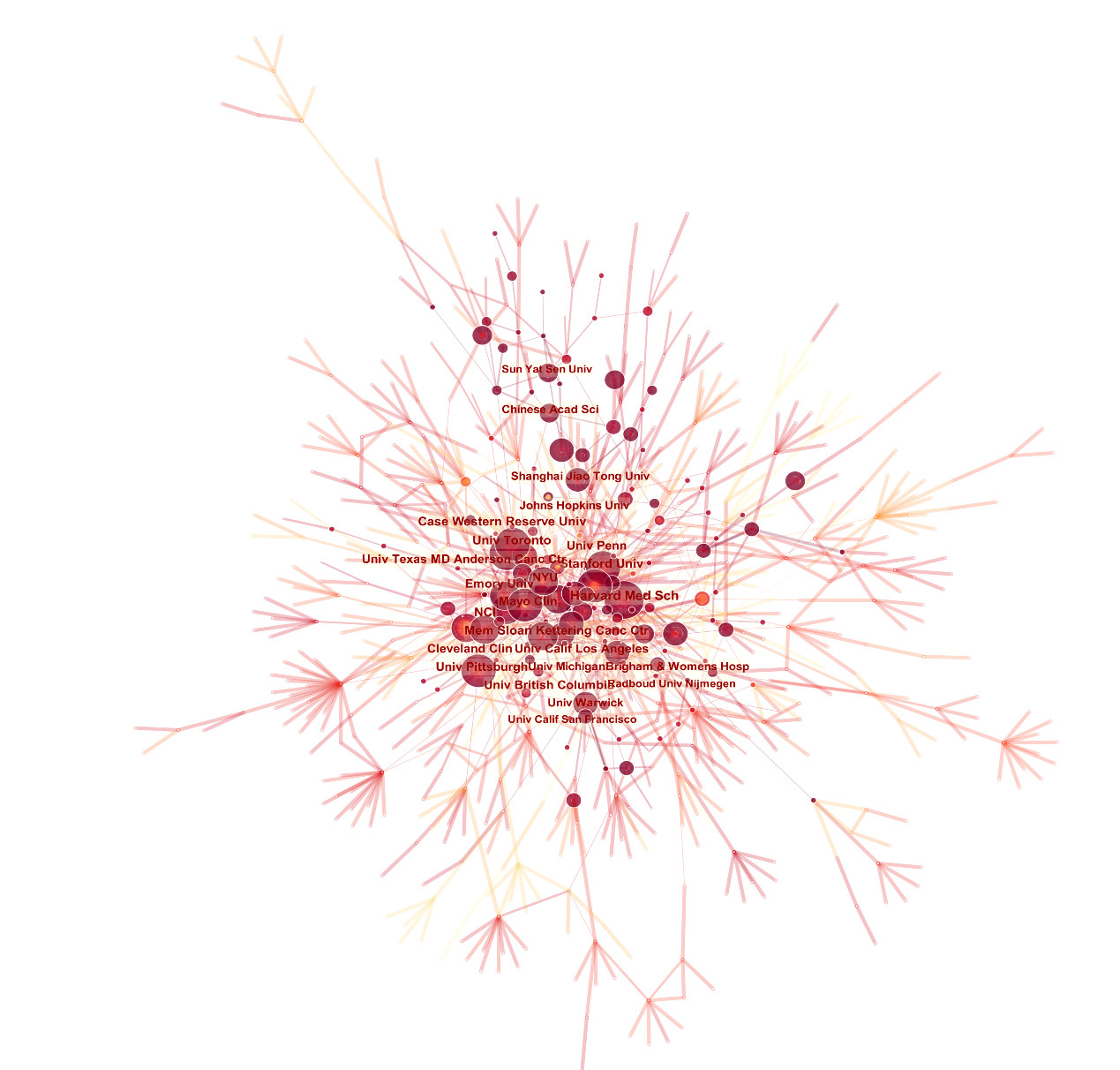
**

**Additional file 1 Figure S1** The institutional cooperation map created with Citespace.

**
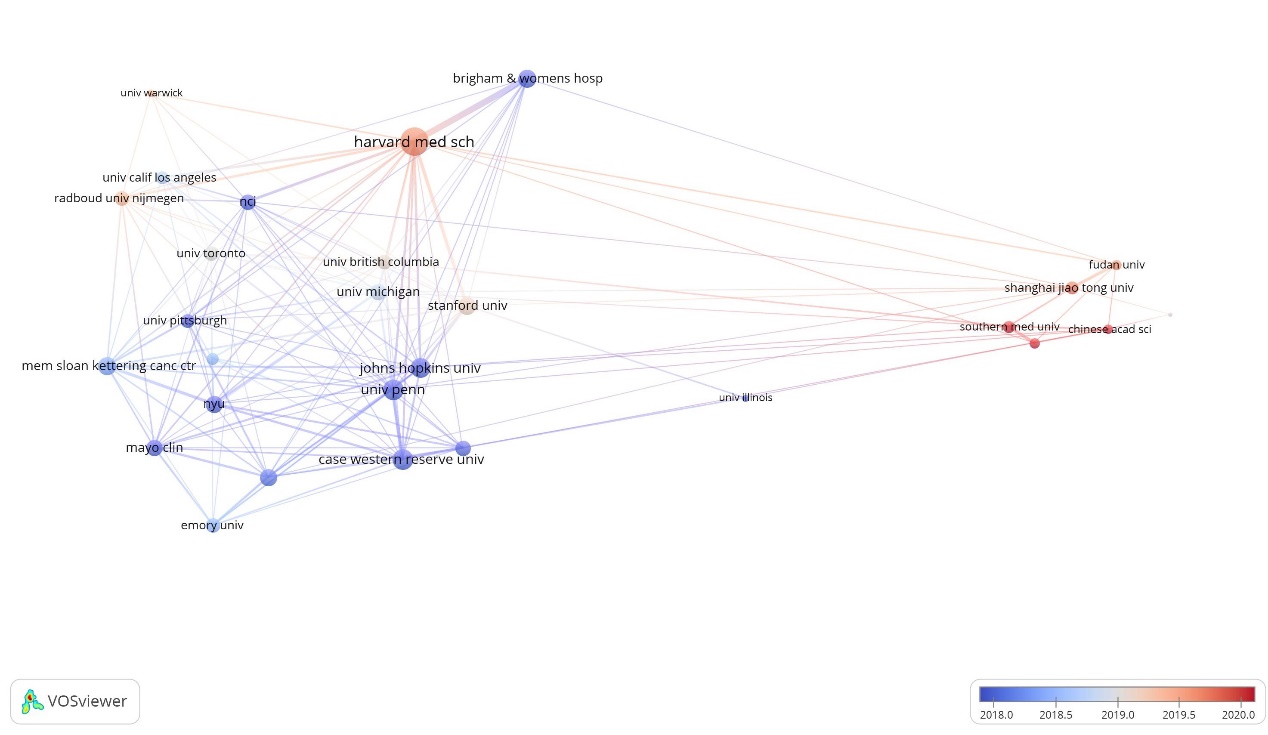
**

**Additional file 1 Figure S2** The overlay visualization map of institution co-authorship analysis generated by VOSviewer.

**Additional file 1 Table S1** The options and settings of VOSviewer for AI-based tumor pathology study.

| Analysis | Unit of analysis | thresholds | Visualization methods |
| --- | --- | --- | --- |
| Co-authorship | Country/region | Minimum number of documents of a country: 20 | Overlay visualization |
|  | Author | Minimum number of documents of an author: 5  Minimum number of citations of an author: 200 | Overlay visualization |
|  | Institution | Minimum number of documents of an institution: 25 | Overlay visualization |
| Co-citation | Journal | Minimum number of documents of a source: 300 | Network visualization |
|  | Cited Reference | Minimum number of citations of a cited reference: 50 | Network visualization |
| Co-occurrence | Author keywords | Minimum number of occurrences of a keyword: 15 | Overlay visualization |

**Additional file 1 Table S2** The top 20 commonly investigated cancers/tumors in the field of AI-based tumor pathology based on the frequency of author keywords co-occurrence.

| Rank | Keywords | Frequency | Rank | Keywords | Frequency |
| --- | --- | --- | --- | --- | --- |
| 1 | breast cancer | 196 | 11 | head and neck cancer | 19 |
| 2 | prostate cancer | 178 | 12 | melanoma | 18 |
| 3 | colorectal cancer | 56 | 13 | colon cancer | 17 |
| 4 | lung cancer | 46 | 14 | endometrial cancer | 17 |
| 5 | gastric cancer | 29 | 15 | hepatocellular carcinoma | 17 |
| 6 | cervical cancer | 24 | 16 | oropharyngeal cancer | 17 |
| 7 | rectal cancer | 24 | 17 | non-small cell lung cancer | 16 |
| 8 | renal cell carcinoma | 23 | 18 | squamous cell carcinoma | 13 |
| 9 | bladder cancer | 19 | 19 | urothelial carcinoma | 13 |
| 10 | glioma | 19 | 20 | kidney cancer | 12 |
